# Comparison of abnormal sensory symptoms in children with and without autism spectrum disorder

**DOI:** 10.12669/pjms.41.4.11486

**Published:** 2025-04

**Authors:** Imran Maqsood, Erum Afzal, Kausar Aftab, Mubashar Ahmad

**Affiliations:** 1 Imran Maqsood, FCPS Department of Developmental & Behavioral Pediatrics, The Children’s Hospital & Institute of Child Health, Multan, Pakistan; 2 Erum Afzal, FCPS Department of Developmental & Behavioral Pediatrics, The Children’s Hospital & Institute of Child Health, Multan, Pakistan; 3 Kausar Aftab, FCPS Department of Developmental & Behavioral Pediatrics, The Children’s Hospital & Institute of Child Health, Multan, Pakistan; 4 Mubashar Ahmad, MS Department of Developmental & Behavioral Pediatrics, The Children’s Hospital & Institute of Child Health, Multan, Pakistan

**Keywords:** Autism spectrum disorder, Hyperresponsiveness, Hyporesponsiveness, Proprioceptive, Sensory

## Abstract

**Objective:**

To compare the abnormal sensory symptoms in children with and without autism spectrum disorder (ASD).

**Method:**

This case-control study was conducted at the department of Developmental & Behavioral Pediatrics, The Children’s Hospital and Institute of Child Health, Multan, Pakistan from January, 2024 to June, 2024. Inclusion criteria for cases were children of either, aged 5-12 years, diagnosed with ASD, and accompanying at least one of the parents. For controls, children visiting outpatient department of pediatrics were included. The study utilized standardized sensory assessment tools “Sensory Profile” to evaluate sensory processing abnormalities in both groups.

**Results:**

In a total of 128 children, 85 (66.4%) were male. The mean age was 8.2±2.4 years, ranging between 5-12 years. In terms of sensory profiles, children with ASD exhibited significantly higher rates of abnormal sensory symptoms across all domains compared to non-ASD children (p<0.001). In the auditory domain, hyperresponsiveness was prevalent in 43.8% of ASD children versus 7.8% in non-ASD children (p<0.001). For tactile, visual, and proprioceptive domains, similar patterns were observed where significantly higher proportions of children with ASD were affected (p<0.001). In the visual and proprioceptive domains, ASD children consistently demonstrated significantly elevated rates of hyperresponsiveness, hyporesponsiveness, and sensory-seeking behaviors compared to non-ASD children (p<0.001).

**Conclusion:**

The high prevalence of hyperresponsiveness, hyporesponsiveness, and sensory-seeking behaviors across sensory domains in ASD children raises the requirement for comprehensive sensory assessments and individualized interventions.

## INTRODUCTION

Autism Spectrum Disorder (ASD) is known to be a neurodevelopmental condition that is characterized by social communication challenges and restricted as well as repetitive behaviors.^1^ According to the “Centers for Disease Control and Prevention (CDC)”, one in 36 children is diagnosed to have ASD in USA.^2^ As sensory processing disorders are expected to affect a large proportion of children with ASD, up to 90% of these children exhibit atypical sensory responses.^3^-^5^ In children, sensory processing is essential in the development and functioning of normal behavior, learning, and social interactions.^6^,^7^ Abnormal sensory symptoms in ASD children may pose significant burden that could affect the individuals as well as their families.^8^ Children with ASD often express a variety of sensory processing abnormalities, including hyperresponsiveness (overreaction to sensory stimuli), hyporesponsiveness (underreaction to sensory stimuli), and sensory seeking (craving intense sensory experiences).^9^,^10^ These sensory abnormalities can manifest in various domains, such as tactile, auditory, visual, and proprioceptive processing. These sensory issues can lead to difficulties in participating in typical childhood activities, such as attending school, playing with peers, and performing daily self-care tasks.^4^,^7^

Despite the high prevalence and significant impact of sensory processing abnormalities in children with ASD, there is a need for comprehensive studies comparing these symptoms in children with and without ASD. Such comparative research can provide insights into the specificity and severity of sensory issues in ASD, guiding the development of tailored interventions and support strategies. This research aimed to enhance our understanding of abnormal sensory symptoms in children with and without ASD. By identifying specific sensory processing patterns and their implications, the study could inform the design of more effective therapeutic approaches, ultimately improving the quality of life for affected children and their families. This research could contribute to early identification and intervention strategies, potentially mitigating the long-term impact of sensory processing issues. The objective of this study was to compare the abnormal sensory symptoms in children with and without ASD.

## METHOD

This case-control study was conducted at the department of Developmental & Behavioral Pediatrics, The Children Hospital and Institute of Child Health Multan from January 2024 to June 2024.

The sample size for this study was calculated using a power analysis to detect a medium effect size (Cohen’s d = 0.5) with a power of 0.80 and an alpha level of 0.05. Based on these parameters, a minimum sample size of 64 children per group (ASD and non-ASD) is required, resulting in a total sample size of 128 participants.

### Ethical Approval:

The study was approved by the Institutional Ethical Committee (Ref. No.: 203/24; dated: January 20, 2024).

### Inclusion criteria:

Children of either, aged 5-12 years, diagnosed with ASD (based on DSM-5 criteria)^11^, and accompanying at least one of the parents. For controls, children visiting outpatient department of pediatrics were included from the outpatient pediatric department, ensuring they had no clinical diagnosis of any neurodevelopmental disorders, including ADHD, developmental delay, or other conditions that may present with sensory processing issues. This was determined based on the medical history provided by the parents during the recruitment process, along with an evaluation by the pediatrician in charge.

### Exclusion criteria:

Children with epilepsy, or severe intellectual disability, or those with significant hearing or vision impairments. For controls, Children with a history or clinical suspicion of any other neurodevelopmental disorders were excluded from the study. Written and informed consents were obtained from parents. Demographic information like gender, age, socio-economic status and medical history were collected. The study utilized standardized sensory assessment tools “Sensory Profile”^12^ to evaluate sensory processing abnormalities in both groups. Parents were asked to complete the Sensory Profile questionnaire based on their observations of their child’s behavior in various sensory domains (e.g., auditory, tactile, visual). For the convenience of local population, the questionnaire was administered in Urdu and for illiterate parents, it was translated into local language by the researchers for recording of responses. Sensory Profile is a 125 items questionnaire that rates items on a Likert scale (e.g., “Always (score=5),” “Frequently (score=4),” “Occasionally (score=3),” “Seldom (score=2),” “Never (score=1)”).

The presence of sensory symptoms such as hyperresponsiveness, hyporesponsiveness, and sensory seeking behaviors was indicated by higher scores (scores>3) on specific items. Hyperresponsiveness was characterized by an exaggerated response to sensory stimuli, as strong reaction to sensory inputs that others might find tolerable or even unnoticed. Auditory hyperresponsiveness was intolerance to loud noises, covering ears, distress in noisy environments. Tactile hyperresponsiveness was discomfort with certain textures, avoidance of touch, overreaction to minor physical contact. Visual hyperresponsiveness was sensitivity to bright lights, avoidance of visually stimulating environments. Proprioceptive hyperresponsiveness was discomfort with certain physical activities or positions, exaggerated responses to body movements.

Hyporesponsiveness referred to a reduced or delayed response to sensory stimuli when the child may seem unresponsive or less aware of sensory inputs that others would typically notice. Auditory hyporesponsiveness was no response to sounds or name being called, seeming unaware of surrounding noises. Tactile hyporesponsiveness was not noticing being touched, high pain threshold, not reacting to temperature changes. Visual hyporesponsiveness was not noticing visual details, lack of reaction to moving objects. Proprioceptive hyporesponsiveness was clumsiness, difficulty understanding body position in space, not noticing when bumped.

Sensory seeking behavior was labeled as children actively seeking out certain sensory experiences. Auditory sensory seeking was labeled as making loud noises, enjoying noisy environments, seeking out sounds. Tactile sensory seeking was described as touching everything, enjoying messy play, seeking out tactile experiences. Visual sensory seeking as staring at bright lights, fascination with spinning objects, seeking visual stimulation. Proprioceptive sensory seeking as Enjoying rough play, seeking deep pressure, engaging in jumping or crashing activities. Socio-economic status was described as low if monthly family income below PKR 18,000, middle if 18,000 to 40,000, or high if above 40,000.^12^

### Statistical analysis:

It was performed using IBM-SPSS Statistics, version 26.0. Mean and standard deviation were calculated for quantitative data. Frequency distributions was calculated for demographics variables and sensory symptoms. Independent sample t-test was utilized to compare the quantitative data whereas categorical variables were compared employing chi-square test. For all inferential statistics, p<0.05 was considered statistically significant.

## RESULTS

In a total of 128 children, 85 (66.4%) were male. The mean age was 8.2±2.4 years, ranging between 5-12 years. Socio-economic status of 56 (43.8%) children was middle while residential status of 67 (52.3%) children was rural. Ninety-two (71.9%) participating parents were mothers. Literacy evaluation of participating parents revealed that 32 (25.0%) were illiterate. Comparison of demographical characteristics of study participants in both study groups showed that no statistically significant differences were observed for age (p=0.6456), gender (p=0.3494), socio-economic status (p=0.9065), residence (p=0.5955), parent participated (p=0.4317), or literacy status of the participating parent (p=0.4142), and the details are shown in Table-I.

**Table-I:** Demographic Characteristics (N=128).

Characteristic		Total (n=128)	ASD (n=64)	Non-ASD (n=64)	P-value
Age (years)		8.2 ± 2.4	8.3 ± 2.3	8.1 ± 2.6	0.6456
Age groups (years)	5-8	62 (48.4%)	32 (50.0%)	30 (46.9%)	0.5984
	9-12	66 (51.6%)	32 (50.0%)	34 (53.1%)	
Gender	Male	85 (66.4%)	45 (70.3%)	40 (62.5%)	0.3494
	Female	43 (33.6%)	19 (29.7%)	24 (37.5%)	
Socio-economic Status	Low	32 (25.0%)	15 (23.4%)	17 (26.6%)	0.9065
	Middle	56 (43.8%)	29 (45.3%)	27 (42.2%)	
	High	40 (31.2%)	20 (31.3%)	20 (31.3%)	
Residence	Rural	67 (52.3%)	35 (54.7%)	32 (50.0%)	0.5955
	Urban	61 (47.7%)	29 (45.3%)	32 (50.0%)	
Parent participating	Mother	92 (71.9%)	48 (75.0%)	44 (68.9%)	0.4317
	Father	36 (20.9%)	16 (25.0%)	20 (31.1%)	
Literacy status of participating parent	Illiterate	32 (25.0%)	14 (21.9%)	18 (28.1%)	0.4142
	Literate	96 (75.0%)	50 (78.1%)	46 (71.9%)	

In terms of sensory profiles, children with ASD exhibited significantly higher rates of abnormal sensory symptoms across all domains compared to non-ASD children (p<0.001), and the details are exhibited in the Table-II. In the auditory domain, hyperresponsiveness was prevalent in 43.8% of ASD children versus 7.8% in non-ASD children (p<0.001). Similar patterns were observed in the tactile, visual, and proprioceptive domains (p<0.001). In the visual and proprioceptive domains, ASD children consistently demonstrated significantly elevated rates of hyperresponsiveness, hyporesponsiveness, and sensory-seeking behaviors compared to non-ASD children (all p < 0.001).

**Table-II:** Comparison of Sensory Symptoms in Children with and without Autism Spectrum Disorder (ASD) Across Different Sensory Domains (N=128).

Domain	Symptom Type	ASD Group (n=64)	Non-ASD Group (n=64)	P-value
Auditory	Hyperresponsiveness	28 (43.8%)	5 (7.8%)	<0.001
	Hyporesponsiveness	22 (34.4%)	4 (6.3%)	<0.001
	Sensory Seeking	14 (21.9%)	4 (6.3%)	<0.001
Tactile	Hyperresponsiveness	31 (48.4%)	6 (9.4%)	<0.001
	Hyporesponsiveness	20 (31.3%)	5 (7.8%)	<0.001
	Sensory Seeking	13 (20.3%)	3 (4.7%)	<0.001
Visual	Hyperresponsiveness	27 (42.2%)	6 (9.4%)	<0.001
	Hyporesponsiveness	24 (37.5%)	5 (7.8%)	<0.001
	Sensory Seeking	13 (20.3%)	4 (6.3%)	<0.001
Proprioceptive	Hyperresponsiveness	30 (46.9%)	5 (7.8%)	<0.001
	Hyporesponsiveness	23 (35.9%)	4 (6.3%)	<0.001
	Sensory Seeking	11 (17.2%)	3 (4.7%)	<0.001

Stratification of gender and age groups across sensory symptoms in children with and without ASD for different sensory domains is shown in Table-III. Children with ASD showed significantly higher sensory abnormalities irrespective of the gender distribution for all domains (p<0.001).

**Table-III:** Stratification of gender across Sensory Symptoms (N=128).

Domain	Symptom Type	Male children				Female children		
		ASD Group (n=45)	Non-ASD Group (n=40)	P-value	ASD Group (n=19)		Non-ASD Group (n=24)	P-value
Auditory	Hyperresponsiveness	20 (44.4%)	4 (10.0)	<0.001	8 (42.1%)		1 (4.2%)	<0.001
	Hyporesponsiveness	15 (33.3%)	3 (7.5%)	<0.001	7 (36.8%)		1 (4.2%)	<0.001
	Sensory Seeking Behaviors	10 (22.2%)	2 (5.0%)	<0.001	4 (21.1%)		2 (8.3%)	<0.001
Tactile	Hyperresponsiveness	22 (48.9%)	5 (12.5%)	<0.001	9 (47.4%)		1 (4.2%)	<0.001
	Hyporesponsiveness	14 (31.1%)	4 (10.0%)	<0.001	6 (31.6%)		1 (4.2%)	<0.001
	Sensory Seeking Behaviors	9 (20.0%)	2 (5.0%)	<0.001	4 (21.1%)		1 (4.2%)	<0.001
Visual	Hyperresponsiveness	21 (46.7%)	4 (10.0%)	<0.001	6 (31.6%)		2 (8.3%)	<0.001
	Hyporesponsiveness	18 (40.0%)	3 (7.5%)	<0.001	6 (31.6%)		2 (8.3%)	<0.001
	Sensory Seeking Behaviors	8 (17.8%)	3 (7.5%)	<0.001	5 (26.3%)		1 (4.2%)	<0.001
Proprioceptive	Hyperresponsiveness	21 (46.7%)	3 (7.5%)	<0.001	9 (47.4%)		2 (8.3%)	<0.001
	Hyporesponsiveness	15 (33.3%)	3 (7.5%)	<0.001	8 (42.1%)		1 (4.2%)	<0.001
	Sensory Seeking Behaviors	8 (17.8%)	2 (5.0%)	<0.001	3 (15.8%)		1 (4.2%)	<0.001

## DISCUSSION

This study revealed that children with ASD exhibited significantly higher rates of hyperresponsiveness, hyporesponsiveness, and sensory-seeking behaviors in auditory, tactile, visual, and proprioceptive domains compared to non-ASD children. Fernandez-Andres MI et al. also investigated sensory processing issues in children with ASD.^13^ They reported that the ASD group obtained scores indicating significant dysfunction across various sensory systems, particularly in social participation and planning and ideas subscales. This research revealed that children with ASD showed higher rates of abnormal sensory symptoms across all domains. In the auditory domain, our finding of 70.3% hyperresponsiveness in ASD children aligns with Kirby AV et al. reporting high percentage of dysfunction in sensory systems, highlighting the pervasive nature of sensory processing issues in ASD. Kirby and colleagues examined the frequency of sensory features in children and found that 74.0% of children had sensory features.^14^

Jussila et al. reported sensory abnormalities in 53.6% of children with ASD, significantly higher than the 8.0% observed in non-ASD children.^15^ Their study also found that tactile hypersensitivity raised the risk for an ASD diagnosis by 34-fold. Our findings are consistent with Jussila et al., as we also observed significantly higher rates of sensory abnormalities in ASD children across all domains. Tactile hyperresponsiveness was reported in 48.4% of ASD children in our study, aligning with Jussila et al. that emphase the prevalence and impact of sensory abnormalities in ASD.^15^ This supports the notion that sensory processing issues are a hallmark of ASD, affecting multiple sensory modalities. Ahn et al.,^16^ and Ben-Sasson et al.,^17^ reported high rates of sensory over-responsivity among general elementary-school-aged children, particularly concerning auditory and tactile sensations. The prevalence of sensory abnormalities was reported to be high among children ASD by Baranek et al.,^18^ and Tomchek and Dunn.^19^ These authors also stated that sensory abnormalities affected 69-95% of children with ASD in clinic-based studies.^18^,^19^ Variation in the proportion of sensory symptoms among various studies could be due the differences in the severity of autistic symptoms, which generally tend to be higher in clinical populations. A local study from Karachi showed that there were definite differences and higher prevalence of sensory symptoms among children with ASD.^20^

The findings from our study and comparisons with existing literature underscore the significant sensory processing challenges faced by children with ASD. The high prevalence of hyperresponsiveness, hyporesponsiveness, and sensory-seeking behaviors across multiple sensory domains in ASD children highlights the need for tailored interventions to address these sensory challenges. Given the high prevalence of sensory abnormalities in ASD, early sensory assessments can help in designing individualized therapeutic strategies. Interventions such as sensory integration therapy, occupational therapy, and environmental modifications can be beneficial in mitigating sensory processing difficulties and improving overall outcomes for children with ASD. Kirby et al.^14^, found that children with sensory features often had co-occurring challenges, including emotional problems, aggression, and hyperactivity. This is consistent with our observation that children with ASD exhibit higher rates of sensory abnormalities, which may contribute to broader behavioral issues. Similarly, Fernandez-Andres et al., noted significant dysfunction in sensory processing related to social participation and planning, aligning with our findings that ASD children struggle with sensory integration across various domains.

Future research should explore the underlying mechanisms contributing to sensory processing abnormalities in ASD. Longitudinal studies can help in understanding the developmental trajectory of sensory processing issues and their impact on various aspects of functioning over time. It is also important to investigate the effectiveness of different intervention strategies in addressing sensory processing issues in ASD. Comparative studies evaluating various therapeutic approaches can inform best practices and guide clinical decision-making. Understanding the role of comorbid conditions, such as anxiety and ADHD, in exacerbating sensory processing difficulties can further refine intervention strategies.

### Limitations

Being a single center study, conducted on a relatively modest sample size, these findings need further verification in large multi-centric trials. Since sensory assessments relied upon parental reports, that could have produced potential reporting bias. Future research should continue to explore the complexities of sensory processing in ASD to inform effective interventions and support strategies.

## CONCLUSION

The high prevalence of hyperresponsiveness, hyporesponsiveness, and sensory-seeking behaviors across sensory domains in ASD children highlights the need for comprehensive sensory assessments and individualized interventions. These findings align with existing literature, underscoring the pervasive nature of sensory processing issues in ASD and the critical need for targeted support to enhance the quality of life and functional outcomes for these children.

### Authors contribution:

**IM:** Data collection and synthesis, responsible for data’s integrity, , critical review.

**EA:** Conception and Idea, Data analysis, critical review, approved the draft for publication.

**KA MA:** Data synthesis, responsible for data’s integrity, critical review, approved the draft for publication.

All authors have approved the final version and are accountable for the integrity of the study.
